# B-Type Natriuretic Peptide as Biomarker of COVID-19 Disease Severity—A Meta-Analysis

**DOI:** 10.3390/jcm9092957

**Published:** 2020-09-12

**Authors:** Sabato Sorrentino, Michele Cacia, Isabella Leo, Alberto Polimeni, Jolanda Sabatino, Carmen Anna Maria Spaccarotella, Annalisa Mongiardo, Salvatore De Rosa, Ciro Indolfi

**Affiliations:** 1 Division of Cardiology and Center of Cardiovascular Research, Department of Medical and Surgical Sciences, Università Magna Graecia di Catanzaro Viale Europa, 88100 Catanzaro, Italy; sabatosorrentino@hotmail.com (S.S.); michele.cacia@gmail.com (M.C.); isabella.leo98@gmail.com (I.L.); polimeni@unicz.it (A.P.); jolesbt@hotmail.it (J.S.); carmenspaccarotella@gmail.com (C.A.M.S.); amongiardo25@gmail.com (A.M.); saderosa@unicz.it (S.D.R.); 2 Mediterranea Cardio Center, 80122 Naples, Italy

**Keywords:** NT-proBNP, death, COVID-19, SARS-CoV-2

## Abstract

Up to 15% of coronavirus disease 2019 (COVID-19) patients experience severe clinical presentation, resulting in acute respiratory distress (ARDS) and finally death. N-terminal natriuretic peptide (NT-proBNP) is associated with a worse prognosis in patients with ARDS. However, whether or not this peptide can help discriminate high-risk COVID-19 patients remains unclear. Therefore, in this meta-analysis, we summarized the available evidence on NT-proBNP in patients admitted for COVID-19. Pooled mean, mean differences (MD) and standardized mean difference (SMD) were the summary metrics. Thirteen studies were finally selected for this analysis with a total of 2248 patients, of which 507 had a severe condition (n = 240) or died (n = 267). Pooled mean NT-proBNP levels on admission were 790.57 pg/mL (95% confidence intervals (CIs): 532.50 to 1048.64) in patients that experienced a severe clinical condition or died, and 160.56 pg/mL (95% CI: 118.15 to 202.96) in non-severe patients (SMD: 1.05; 95% (CI): 0.83 to 1.28; *p* < 0.001; I^2^ 74%; and MD was 645.84 pg/mL (95% CI: 389.50–902.18). Results were consistent in studies categorizing patients as non-survivors versus survivors (SMD: 1.17; 95% CI 0.95 to 1.40; *p* < 0. 001; I^2^: 51%), and in those classifying populations in severe versus non-severe clinical condition (SMD: 0.94 95% CI 0.56 to 1.32; *p* < 0.001; I^2^: 81%; *p*_interaction_ = 0.30). In conclusion, our results suggest that assessing NT-proBNP may support physicians in discriminating high-risk COVID-19 patients.

## 1. Introduction

The outbreak of coronavirus disease 2019 (COVID-19) caused by severe acute respiratory syndrome coronavirus 2 (SARS-CoV-2) is affecting a large number of countries and territories around the world. Up to 15% of the affected patients experience acute respiratory distress syndrome (ARDS), multiple organ dysfunction, and finally death; thus challenging healthcare delivery and related costs [1]. Usual markers for congestive heart failure, B-type natriuretic peptide (BNP), and its N-terminal portion (NT-proBNP), are also associated with an unfavorable course among patients with ARDS [2]. Accordingly, this peptide may theoretically be used as an indicator of clinical severity for SARS-CoV-2 infection. For instance, both the involvement of the respiratory system and the use of the mechanical ventilator support may increase pulmonary vascular tone, causing right ventricular afterload and wall stretching; representing the strongest mechanical stimuli provoking BNP release [3]. Furthermore, recently published observations documented a direct involvement of the myocardium in COVID-19 patients linking increasing levels of NT-proBNP with mortality [4]. However, current evidence lacks large studies evaluating the prognostic value of NT-proBNP in COVID-19 patients, and a routinely and longitudinal assessment of this peptide is still not recommended [5]. Hence, we reviewed current scientific literature to investigate whether the measurement of NT-proBNP may help discriminate clinical severity in patients with COVID-19.

## 2. Experimental Section

This meta-analysis has been performed according to the Preferred Reporting Items for Systematic Reviews and Meta-Analyses (PRISMA) guidelines [6] (Supplementary Table S1). We searched MEDLINE, Scopus, and Google Scholar for the latest papers published or posted before 7 August 2020. The following keywords were used for the search: “laboratory” or “NT-proBNP” or “BNP” and “coronavirus 2019” or “2019-nCoV” or “SARS-CoV-2”. We selected full-length manuscripts, without language restrictions, in which NT-proBNP has been assessed in COVID-19 patients, on admission. Moreover, we included studies categorizing patients according to the severity of the clinical condition (severe versus non-severe) (Supplementary Table S2) or survival status (non-survivors and survivors). The main exclusion criteria were editorials, letters, expert opinions, case reports or series, studies with duplicated data or less than a total of 50 patients or 15 patients in one of the two groups. Furthermore, studies with a significant number of missing values, or that did not clearly state on admission assessment of NT-proBNP were also excluded in this analysis. Pre-specified data elements were extracted from each study and included in a structured dataset. Furthermore, mean pooled NT-proBNP levels were calculated for each group, as well as the main difference (MD) and their respective 95% confidence intervals (CIs), as well as standardized mean difference (SMD). The primary analytic method was random-effect models. Heterogeneity among studies was estimated with chi-square tests and quantified with I^2^ statistics. Publication bias was assessed using visual estimation of a funnel plot. Risk of bias was assessed using Agency for Healthcare Research and Quality guidelines, as previously described [7]. When not reported, mean and standard deviation values were derived from the sample size, median and interquartile range (IQR), as previously described [8,9]. Statistical analyses were performed with Open Meta-Analyst (Brown University; Providence, RI, USA) and RevMan, software version 5.3 (The Cochrane Collaboration; Copenhagen, Denmark).

## 3. Results

**Figure 1:**
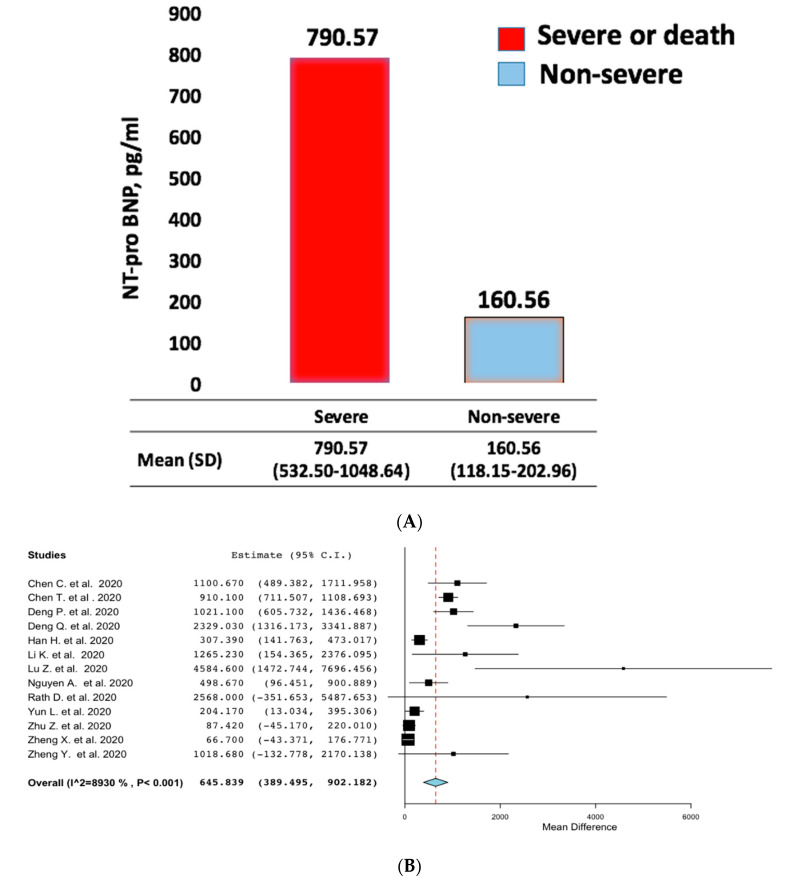
Pooled (**A**) and mean difference (**B**) of N-terminal natriuretic peptide (NT-proBNP) levels in severe or death and non-severe patients.

Out of 513 screened reports for eligibility, thirteen were finally included in this analysis (Supplementary Figure S1 and Supplementary Table S3). The baseline characteristics of the studies were summarized in Table 1. Out of 2248 patients, 507 had a severe clinical condition (n = 240) or died (n = 267). Patients with a severe clinical condition were older, with a lower prevalence of female sex. The time between symptom onset and hospital admission was similar across the study groups. Pooled mean NT-proBNP levels were 790.57 pg/mL (95% CIs: 532.50 to 1048.64) in non-survivors or severe patients, and 160.56 pg/mL (95% CI: 118.15 to 202.96) in survivors or patients not experiencing a severe clinical condition (Figure 1A), with a MD of 645.84 pg/mL (95% CI: 389.50–902.18) (Figure 1B) and a SMD: 1.05 (95% CI 0.83 to 1.28; *p* < 0.001; I^2^: 74%) (Supplementary Figure S2). No evidence of bias was observed (Supplementary Figure S3, Supplementary Table S4). Finally, results were consistent among non-survivors versus survivors (SMD: 1.17 95% CI 0.95 to 1.40; *p* < 0.001; I^2^: 51%), and among patients that experienced a severe versus non-severe clinical condition (SMD: 0.94 95% CI 0.56 to 1.32; *p* < 0.001; I^2^: 81%; *p*_interaction_ = 0.30) (Supplementary Figure S1).

## 4. Discussion

The major finding of the present meta-analysis, including 13 observational studies and a total of 2248 patients, is that an elevated NT-proBNP level on admission is associated with a worse prognosis in COVID-19 patients. Previous evidence has shown that BNP plays a key role in the pathophysiology of heart failure and that NT-Pro-BNP is a widely confirmed disease severity biomarker [10,11]. Several reasons may lead to the release of NT-proBNP in patients with pneumonia. First, hypoxia-induced pulmonary hypertension as well as the use of vasopressor agents in critically ill patients may increase myocardial wall stress and contribute to the increasing levels of NT-proBNP. Second, direct involvement of the myocardium tissue by the activation of the inflammatory system, oxidative stress, and demand-supply mismatch, or by a direct virus-induced myocardial invasion and injury may also cause the NT-proBNP release. Third, the occurrence of renal failure that may also increase NT-proBNP levels by impairing its clearance [12]. Therefore, it will be interesting to see whether a reduction in NT-proBNP levels indicates a favorable course of the disease among COVID-19 patients. However, its prognostic value among patients with SARS-CoV-2 infection is uncertain. In a retrospective analysis by Guo et al., including 187 patients with confirmed COVID-19, NT-proBNP levels increased significantly during hospitalization only for patients who died, while such significant dynamic changes were not present among survivors [1]. Similarly, in an analysis including 416 hospitalized patients with COVID-19, NT-BNP was significantly higher among patients with myocardial injury compared with those without. However, in this population, NT-proBNP was not an independent predictor of mortality [4]. In our meta-analysis, 507 patients (22.8% of the entire population) died or experienced severe clinical conditions, with a six-fold increase in NT-proBNP levels over the upper reference limit at admission (mean 790.57, 95% CI 532.50 to 1048.64 pg/mL). Although the high accuracy of NT-proBNP is already established in the diagnosis of acute heart failure, the prognostic value of this marker for patients with COVID-19 remains uncertain. However, in this case, which presents more diagnostic and therapeutic indecision, assessing NT-proBNP certainly provides key information, integrating routinely performed clinical and analytical markers. In particular, such a high level of NT-proBNP may suggest clinicians admit the patient or perform a more accurate cardiac evaluation, to exclude a direct or indirect myocardial involvement. Furthermore, assessing NT-proBNP may represent a surrogate of invasive monitoring in a context of a poor resource setting and support the tailoring of medical therapy.

## 5. Conclusions

In our analysis, we aimed to summarize previous evidence looking at the relationship between NT-proBNP levels assessed on admission and the severity of COVID-19 disease. Of note, we observed that NT-proBNP levels at admission were increased in critically ill COVID-19 patients or in those who died compared to non-severe patients; an effect that was consistent in both subgroups of patients (severe vs. non-severe; non-survivors vs. survivors). However, since our results were derived from observational studies, further investigations are warranted to confirm whether or not NT-proBNP should be routinely used as a prognostic marker for patients admitted with the diagnosis of COVID-19.

## Figures and Tables

**Table 1:** Baseline studies’ characteristics.

	**Chen C. et al.**	**Chen T. et al.**	**Deng P. et al.**	**Deng Q. et al.**	**Han H. et al.**	**Li K. et al.**	**Lu Z. et al.**
**Journal**	JCMA	BMJ	CCA	IJC	JMV	MedRxiv	Lancet
**Study Design**	Retrospective, multicenter, single arm registry	Retrospective, single center, single arm registry	Retrospective, single center, single arm registry	Retrospective, single center, single arm registry	Retrospective, single center, single arm registry	Retrospective, single center, single arm registry	Retrospective, single center, single arm registry
**Enrollment Period**	January–February 2020	13 January–12 February 2020	January–February 2020	6 January–20 February 2020	1 January–18 February 2020	31 January–5 March 2020	January–February 2020
**Groups definition**	Severe vs. non-severe	Non-Survivors vs. survivors	Non-Survivors vs. survivors	Severe vs. non-severe	Severe vs. non-severe	Non-Survivors vs. survivors	Non-Survivors vs. survivors
**Death or Severe group**	24	113	52	67	60	15	31
**Age**	68.5 (13.6)	68.0 (62.0–77.0)	74.5 (65.3–81.8)	68.0 (57.0–77.0)	59.1 (14.4)	69.0 (58.0–77.0)	72.0 (9.0)
**Female (%)**	6 (25.0%)	30 (26.6%)	19 (36.5%)	29 (43.3%)	39 (65.0%)	4 (27.0%)	9 (29.1%)
**Days from symptom onset to hospital admission**	NA	10 (7–13)	NA	NA	NA	9 (6–14)	22.1 (17.0–28.0)
**NT pro BNP (pg/mL)**	1030.0 (339.0–2276.0)	800.0 (389.8–1817.5)	943.2 (402.3–2.397,5)	1142.0 (388.3–5956.5)	290.9 (106.1–958.1)	817.5 (348.5–3031.0)	4868.0 (8839.0)
**EF (%)**	NA	NA	NA	58.5 ± 5.4	NA	NA	NA
**Non-severe group**	126	161	212	45	198	87	92
**Age**	57.1 (15.6)	51.0 (37.0–66.0)	62.5 (52.0–70.0)	56.0 (39.0–67.0)	59.0 (10.8)	55.0 (44.0–66.0)	53.0 (14.0)
**Female (%)**	60 (47.6%)	73 (45.4%)	115 (54.2%)	26 (57.8%)	127 (64.2%)	39 (44.8%)	53 (57.6%)
**Days from symptom onset to hospital admission**	NA	9.0 (6.0–12.0)	NA	NA	NA	11.0 (8.0–18.0)	17.7 (13.0–23.0)
**NT pro BNP (pg/mL)**	83.0 (28.0–232.0)	72.0 (20.0–185.0)	155.0 (64.4–460.3)	101.9 (34.0–363.8)	113.7 (45.9–274.2)	92.5 (42.3–266.5)	283.4 (229.1)
**EF (%)**	NA	NA	NA	62.0 ± 5.5	NA	NA	NA
	**Nguyen A.B. et al.**	**Rath D. et al.**	**Yun L. et al.**	**Zhu Z.**	**Zheng X. et al.**	**Zheng Y. et al.**	
**Journal**	MedRxiv	CRIC	CJID	IJID	Lancet	JCV	
**Study Design**	observational single center, single arm registry	prospective, single center, single arm registry	Retrospective, single center, single arm registry	Retrospective, single center, single arm registry	Retrospective, single center, single arm registry	Retrospective, single center, single arm registry	
**Enrollment Period**	16 March–16 April 2020	1 February–31 March 2020	20 January–10 February 2020	23 January–20 February 2020	January–March 2020	16 January–20 February 2020	
**Groups definition**	Non-Survivors vs. survivors	Non-Survivors vs. survivors	Severe vs. non-severe	Severe vs. non-severe	Severe vs. non-severe	Critical vs. non critical	
**Severe group**	45	16	21	16	22	32	
**Age**	72.0 (61.0–81.0)	73.0 (16.0)	65.5 (15.7)	57.5 (11.70)	58.2 (13.2)	63.8 (16.5)	
**Female (%)**	20 (44.4%)	4 (25.0%)	2 (9.5%)	7 (43.8%)	11 (50.0%)	NA	
**Days from symptom onset to hospital admission**	NA	NA	5 (3–7)	6.9±2.8	4.6 ± 3.7	NA	
**NT pro BNP (pg/mL)**	407.0 (109–1.779)	1992.0 (416–7719)	95.1 (39.2–601.2)	196.5 (75.9–405.1)	180.2 (209.8)	1085.5 (3217.1)	
**EF (%)**	NA	49.0 (12.1)	NA	NA	NA	NA	
**Non-severe group**	308	107	271	111	30	67	
**Age**	60.0 (48.5–71.5)	67.0 (15.0)	48.7 (15.7)	50.0 (15.5)	45.6 (16.0)	42.5 (15.1)	
**Female (%)**	164 (53.2%)	42 (39.3%)	136 (50.2%)	38 (34.2%)	18 (60%)	NA	
**Days from symptom onset to hospital admission**	NA	NA	4.0 (2.0–7.0)	5.1 (3.8)	5.7 (6.0)	NA	
**NT pro BNP (pg/mL)**	112.0 (29.0–658.0)	377 (132.0–1914.0)	34.7 (23.3–65.1)	118.0 (78.4–218.8)	113.5 (186.0)	66.9 (90.9)	
**EF (%)**	NA	58.0 ± 6.0	NA	NA	NA	NA	

Data are reported as N and percentage (%), or median and interquartile ranges (IQRs) or mean and ± standard deviation (SD) when appropriate. JCMA: Journal of Chinese Medical Association; BMJ: British Medical Journal; IJC: International Journal of Cardiology; JMV = Journal of Medical Virology; CJID: Chinese Journal of Infectious Disease. CCA: Clinica Chimica Acta. CRIC: Clinical Research in Cardiology. JPU: Journal of Peking University. JCV: Journal of Clinical Virology, IJID: International Journal of Infectious Diseases. Study definition of severity are reported in the supplementary Table S2; NT-proBNP: N-terminal prohormone B-type natriuretic peptide; NA: not available.

## Supplementary Materials

The following are available online at https://www.mdpi.com/2077-0383/9/9/2957/s1, Supplementary Table S1: PRISMA checklist, Supplementary Table S2: Severity criteria, Supplementary Table S3: List of included articles, Supplementary Table S4: Quality assessment table according to Agency for Healthcare Research and Quality (AHRQ) guidelines, Supplementary Figure S1: PRISMA 2009 Flow Diagram; Pooled means (A) and mean differences (B) for NT-proBNP in COVID-19 patients with severe or non-severe clinical presentation. SD: Standard deviation, Supplementary Figure S2: Standardized mean difference and 95% confidence interval of natriuretic peptides values between patients with or without severe form of COVID-19, Supplementary Figure S3: Funnel plot analysis of NT-proBNP values between patients with or without severe form of coronavirus disease 2019.
