# Supplementary material for: B-Type Natriuretic Peptide as Biomarker of COVID-19 Disease Severity—A Meta-Analysis
Source: J Clin Med. 2020 Sep 12;9(9):2957. doi: 10.3390/jcm9092957 (PMC7564464; doi:10.3390/jcm9092957)
Supplement: Supplementary file 1 [file jcm-09-02957-s001.pdf]

Supplementary Table S1. PRISMA checklist.

Section/topic	#	Checklist item	Reported on page #
		TITLE	
Title	1	Identify the report as a systematic review, meta-analysis, or both.	1
		ABSTRACT	
Structured summary	2	Provide a structured summary including, as applicable: background; objectives; data sources; study eligibility criteria, participants, and interventions; study appraisal and synthesis methods; results; limitations; conclusions and implications of key findings; systematic review registration number.	2
		INTRODUCTION	
Rationale	3	Describe the rationale for the review in the context of what is already known.	3
Objectives	4	Provide an explicit statement of questions being addressed with reference to participants, interventions, comparisons, outcomes, and study design (PICOS).	3
		METHODS	
Protocol and registration	5	Indicate if a review protocol exists, if and where it can be accessed (e.g., Web address), and, if available, provide registration information including registration number.	NA
Eligibility criteria	6	Specify study characteristics (e.g., PICOS, length of follow-up) and report characteristics (e.g., years considered, language, publication status) used as criteria for eligibility, giving rationale.	3
Information sources	7	Describe all information sources (e.g., databases with dates of coverage, contact with study authors to identify additional studies) in the search and date last searched.	3
Search	8	Present full electronic search strategy for at least one database, including any limits used, such that it could be repeated.	3
Study selection	9	State the process for selecting studies (i.e., screening, eligibility, included in systematic review, and, if applicable, included in the meta-analysis).	3
Data collection process	10	Describe method of data extraction from reports (e.g., piloted forms, independently, in duplicate) and any processes for obtaining and confirming data from investigators.	3-4
Data items	11	List and define all variables for which data were sought (e.g., PICOS, funding sources) and any assumptions and simplifications made.	3-4
Risk of bias in individual studies	12	Describe methods used for assessing risk of bias of individual studies (including specification of whether this was done at the study or outcome level), and how this information is to be used in any data synthesis.	4
Summary measures	13	State the principal summary measures (e.g., risk ratio, difference in means).	4
Synthesis of results	14	Describe the methods of handling data and combining results of studies, if done, including measures of consistency (e.g., I^2) for each meta-analysis.	4

**Risk of bias across studies**	**15**	**Specify any assessment of risk of bias that may affect the cumulative evidence (e.g., publication bias, selective reporting within studies).**	**4**
**Additional analyses**	**16**	**Describe methods of additional analyses (e.g., sensitivity or subgroup analyses, meta-regression), if done, indicating which were pre-specified.**	**4**
		**RESULTS**	
**Study selection**	**17**	**Give numbers of studies screened, assessed for eligibility, and included in the review, with reasons for exclusions at each stage, ideally with a flow diagram.**	**4**
**Study characteristics**	**18**	**For each study, present characteristics for which data were extracted (e.g., study size, PICOS, follow-up period) and provide the citations.**	**4**
**Risk of bias within studies**	**19**	**Present data on risk of bias of each study and, if available, any outcome level assessment (see item 12).**	**4**
**Results of individual studies**	20	**For all outcomes considered (benefits or harms), present, for each study: (a) simple summary data for each intervention group (b) effect estimates and confidence intervals, ideally with a forest plot.**	**4**
**Synthesis of results**	**21**	**Present results of each meta-analysis done, including confidence intervals and measures of consistency.**	**4**
**Risk of bias across studies**	**22**	**Present results of any assessment of risk of bias across studies (see Item 15).**	**4**
**Additional analysis**	**23**	**Give results of additional analyses, if done (e.g., sensitivity or subgroup analyses, meta-regression [see Item 16]).**	**4**
		**DISCUSSION**	
**Summary of evidence**	**24**	**Summarize the main findings including the strength of evidence for each main outcome; consider their relevance to key groups (e.g., healthcare providers, users, and policy makers).**	**4**
**Limitations**	**25**	**Discuss limitations at study and outcome level (e.g., risk of bias), and at review-level (e.g., incomplete retrieval of identified research, reporting bias).**	**5**
**Conclusions**	**26**	**Provide a general interpretation of the results in the context of other evidence, and implications for future research.**	**5**
		**FUNDING**	
**Funding**	**27**	**Describe sources of funding for the systematic review and other support (e.g., supply of data); role of funders for the systematic review.**	**NA**

NA: not available.

Supplementary Table S2: Severity criteria.

Study	Criteria for disease severity
Chen C. et al	One of the following conditions:<br>- respiratory failure and mechanical ventilation required;<br>- shock;<br>- admission to the intensive care unit (ICU) for treatment
Deng Q. et al	Severe (one of the three criteria: respiratory distress and respiratory rate higher than 30 times per minute; fingertip blood oxygen saturation <93% at rest; partial arterial oxygen pressure (PaO_2)/fraction of inspiration oxygen (FiO_2) < 300 mmHg) plus critical type (one of three conditions: respiratory failure, requiring mechanical ventilation; shock; multiple organ failure, requiring intensive care management)
Han H. et al	Severe cases (at least one of the following conditions: (a) shortness of breath, RR ≥ 30 times/min, (b) oxygen saturation (resting state) ≤93%, or (c) PaO2/FiO2 ≤ 300mm Hg) plus Critical cases (at least one of the extra following conditions: (a) respiratory failure that needs to receive mechanical ventilation; (b) shock; and (c) multiple organ failure that need to be transferred to the intensive care unit (ICU)
Yun L. et al	Severe Pneumonia (patients with the following severe manifestations: fever or suspected respiratory infection, plus one of a respiratory rate >30 breaths/min, severe respiratory distress, or SpO2 <90% on room air. Patients with ARDS, sepsis, or septic shock were also included.
Zhe Z. et al	Severe patients should meet at least one of the following criterions: First, shortness of breath with respiration rate (RR) ≥30 times/min. Second, oxygen saturation ≤93% in resting state. Third, partial pressure of arterial oxygen (PaO2)-to-fraction of inspired oxygen (FiO2) ratio ≤300 mm Hg.<br>Obvious lesion progression >50% within 24-48 hours on pulmonary imaging were also recognized as severe cases.<br>Critical cases were defined when one of the following conditions met: First, respiratory failure and require mechanical ventilation. Second, shock occurred. Third, combined with other organ failure and treated in intensive care unit.<br>Mild and moderate cases were defined as non-severe group, while severe and critical patients were categorized as severe group in this study.
Zheng X. et al	Severe type: any of the following:<br>- Shortness of breath, breathing frequency ≥30 times /min;<br>- At rest, oxygen saturation ≤93%;<br>-Arterial partial pressure (PaO2)/oxygen absorption concentration (FiO2) ≤300 mmHg.
Zheng Y. et al	Critical cases (any of the following criteria: (1) Increased breathing rate (≥ 30 beats/min), difficulty breathing or cyanosis of the lips; (2) upon inhalation, oxygen saturation was ≤ 93 %; (3) arterial blood oxygen partial pressure (PaO2)/oxygen concentration (FiO2) was ≤300 mmHg (1 mmHg = 0.133 kPa); (4) pulmonary imaging showed multilobular lesions or lesion progression within 50 h of > 50 %; or (5) other clinical conditions requiring hospitalization) and severe critical cases (any one of the following conditions: (1) respiratory failure occurred, requiring mechanical ventilation; (2) shock occurred; or (3) ICU monitoring and treatment were required for combined organ failure) vs ordinary cases

Supplementary Table S3: List of included articles.

	Article	Peer-review
**1**	Analysis of myocardial injury in patients with COVID-19 and association between concomitant cardiovascular diseases and severity of COVID-19. Chen C, Chen C, Yan JT, Zhou N, Zhao JP, Wang DW. Journal of Chinese Medical Association. 2020 Mar 6;48(0):E008. doi:10.3760/cma.j.cn112148-20200225-00123.	**Yes**
**2**	Clinical characteristics of 113 deceased patients with coronavirus disease 2019: retrospective study. Chen T., Wu D., Chen H. Yan W., Yang D., Chen G., Ma K., Xu D., Yu H., Wang H., Wang T., Guo W., Chen J., Ding C., Zhang X., Huang J., Han M., Li S., Luo X., Zhao J., Ning Q. BMJ 2020; 368. doi: https://doi.org/10.1136/bmj.m1091	**Yes**
**3**	Suspected myocardial injury in patients with COVID-19: Evidence from front-line clinical observation in Wuhan, China. Deng Q., Hu B., Zhang Y., Wang H., Zhou X., Hu W., Cheng Y., Yan J., Ping H., Zhou Q. Int J Cardiol. 2020 Apr 8. doi: 10.1016/j.ijcard.2020.03.087.	**Yes**
**4**	Analysis of heart injury laboratory parameters in 273 COVID-19 patients in one hospital in Wuhan, China. Han H., Xie L., Liu R., Yang J., Liu F., Wu K., Chen L., Hou W., Feng Y., Zhu C. J Med Virol. 2020 Mar 31. doi: 10.1002/jmv.25809.	**Yes**
**5**	Radiographic Findings and other Predictors in Adults with Covid-19. Li K., Chen D., Chen S., Feng Y., Chang C., Wang Z., Wang N., Zhen G. MedRxiv (March 27, 2020). doi: https://doi.org/10.1101/2020.03.23.20041673.	**No**
**6**	Clinical Characteristics And Risk Factors For Fatal Outcome in Patients With 2019-Coronavirus Infected Disease (COVID-19) in Wuhan, China. Lu Z., Chen M., Fan Y., Wu X. Zhang L., Guo T., Deng K., Cao J., Luo H., He T., Gong Y., Wang H., Wan J., Wang X., Lu Z. The Lancet. Online Preprint 2/27/2020. doi: http://dx.doi.org/10.2139/ssrn.3546069.	**No**
**7**	Clinical analysis on risk factors for COVID-19 patients becoming severe patients. Yun L., Gian Z., Huang D., Zhang D., Li T., Liu M., Song S., Wang J., Zhang Y., Xu S., Chen J., Zhang J., Zhu T., Hu B., Wang S., Mao E., Zhu L., Zhang W., Lu H. Chinese Journal of Infectious Diseases, 2020, 38; Online Preprint. doi: 10.3760/cma.j.cn311365-20200211-00055.	**Yes**
**8**	Clinical Features and Risk Factors for the Severity of Inpatients with COVID-19: A Retrospective Cohort Study. Zheng X., Chen J., Deng L., Fang Z., Chen G., Ye D., Hong Z., Xia J. The Lancet. Online Preprint 3/24/2020. doi: http://dx.doi.org/10.2139/ssrn.3562460	**No**
**9**	The diagnostic and prognostic role of myocardial injury biomarkers in hospitalized patients with COVID-19. Deng P., Ke Z., Ying B., Qiao B, Yuan L. Clinica Chimica Acta. 2020 Jul 16. doi: https://doi.org/10.1016/j.cca.2020.07.018	**Yes**
**10**	Outcomes and cardiovascular comorbidities in a predominantly africanamerican population with covid-19. Nguyen A.B., Upadhyay G.A., Chung B., Smith B., Besser MSAS S.A., Johnson J.A., Blair J., Parker Ward R., DeCara J., Polonsky T., Patel A.R., Grinstein J., Holzhauser L., Kalathiya R., Shah A.P., Paul J., Nathan S., Liao J, Lang R.M., Wolfe K., Adegunsoye A., Wu D., Patel B., Peek M.E., Miller D., Kurian D.J., Estime S.R., Dalton A., Tung A., O'Connor M.F., Kress J.P., Alenghat F.J., Tung R. MedRxiv (June 29,2020). doi: https://doi.org/10.1101/2020.06.28.20141929	**No**
**11**	Impaired cardiac function is associated with mortality in patientswith acute COVID-19 infection. Rath D., Petersen-Uribe A., Avdiu A., Witzel K., Jaeger P., Zdanyte M., Heinzmann D., Tavlaki E., Muller K., Gawaz M.P. Clinical Research in Cardiology (May 28, 2020). doi: https://doi.org/10.1007/s00392-020-01683-0	**Yes**
**12**	Epidemiological characteristics and clinical features of 32 critical and 67 noncritical cases of COVID-19 in Chengdu. Zheng Y., Xu H., Yang M., Zeng Y., Chen H., Liu R., Li O., Zhang N., Wang D. Journal of Clinical Virology. (April 5, 2020). doi: https://doi.org/10.1016/j.jcv.2020.104366	**Yes**
**13**	Clinical value of immune-inflammatory parameters to assess the severity of coronavirus disease 2019 Zhe Zhu, Ting Cai, Lingyan Fan, Kehong Lou, Xin Hua, Zuoan Huang, Guosheng Gaodoi: 10.1016/j.ijid.2020.04.041.	

Supplementary Table S4: Quality assessment table according to Agency for Healthcare Research and Quality (AHRQ) guidelines.

Study	Chen C. et al.	Chen T. et al.	Deng P. et al	Deng Q. et al	Han H. et al.	Li K. et al.	Lu Z. et al.	Nguyen AB et al	Rath D. et al.	Yun L. et al	Zhu Z. et al	Zheng X. et al.	Zheng Y. et al.
Define the source of information	Yes	Yes	Yes	Yes	Yes	Yes	Yes	Yes	Yes	Yes	Yes	Yes	Yes
List inclusion and exclusion criteria for exposed and unexposed subjects or refer to previous publications	Yes	Yes	Yes	Yes	Yes	Yes	Yes	Yes	Yes	Yes	Yes	Yes	Yes
Indicate time period used for identifying patients	Yes	Yes	Yes	Yes	Yes	Yes	Yes	Yes	Yes	Yes	Yes	Yes	Yes
Indicate whether or not subjects were consecutive if not population-based	Unclear	Unclear	Unclear	Unclear	Unclear	Unclear	Yes	Yes	Yes	Unclear	Yes	Unclear	Yes
Indicate if evaluators of subjective components of study were masked to other aspect of the status of participants	Unclear	Unclear	No	Unclear	Unclear	Unclear	Unclear	No	No	Unclear	No	Unclear	No
Describe any assessments undertaken for quality assurance purposes	No	Yes	Yes	Yes	No	Yes	Yes	No	No	No	No	Yes	No
Explain any patient exclusions from analysis	No	No	Yes	Yes	No	Unclear	Yes	Yes	No	No	Yes	Yes	No
Describe how confounding was assessed and/or controlled.	Yes	No	Yes	Yes	No	Yes	Yes	Yes	No	No	Yes	Yes	No
If applicable, explain how missing data were handled in the analysis	No	No	Yes	No	No	No	Yes	No	No	No	No	No	No
Summarize patients response rates and completeness of data collection	No	Yes	No	Yes	No	No	No	No	No	No	No	Yes	No
Clarify what follow up, if any was expected and the percentage of patients for which incomplete data or follow up was obtained.	No	No	Yes	No	No	No	No	No	Yes	No	No	No	Yes

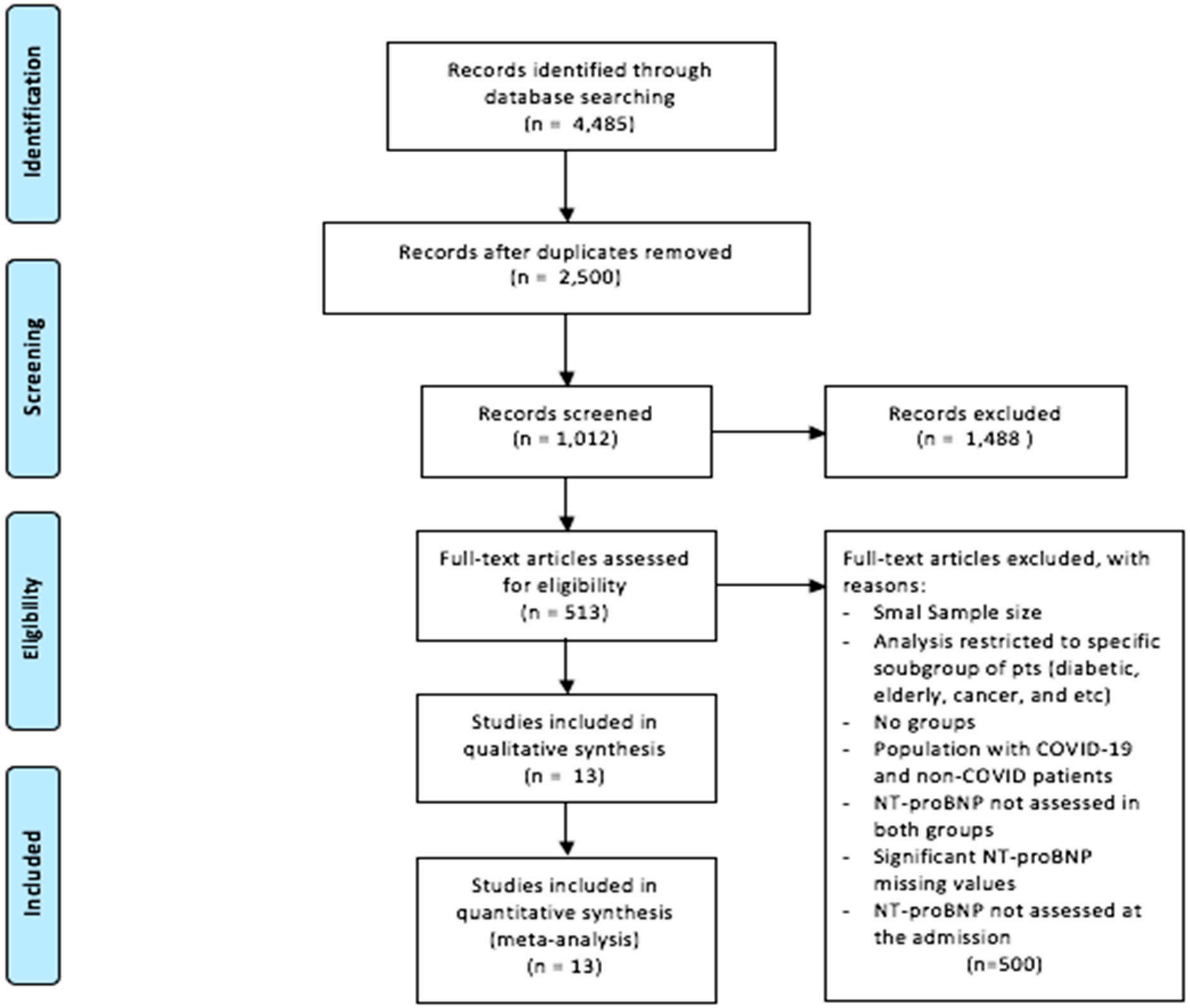

Supplementary Figure S1: PRISMA 2009 Flow Diagram; Pooled means (A) and mean differences (B) for NT-proBNP in COVID-19 patients with severe or non-severe clinical presentation. SD: Standard deviation.

Study or Subgroup	Severe or death Mean	SD	Total	Non-severe Mean	SD	Total	Weight	Std. Mean Difference IV, Random, 95% CI
**1.7.1 NT-proBNP severe vs. non severe**								
Chen C. et al.	1,215	1,526.47	24	114.33	152.99	126	7.2%	1.77 [1.29, 2.25]
Deng Q. et al.	2,495.6	4,218.73	67	166.57	252.56	45	8.2%	0.71 [0.32, 1.10]
Han H. et al.	451.99	647.81	60	144.6	170.5	198	9.1%	0.89 [0.59, 1.19]
Yun L. et al.	245.17	446.81	21	41	31.15	271	7.4%	1.68 [1.22, 2.15]
Zheng X. et al.	180.2	209.8	22	113.5	186	30	6.5%	0.33 [-0.22, 0.89]
Zheng Y. et al.	1,085.6	3,217.1	30	66.92	90.85	54	7.5%	0.53 [0.07, 0.98]
Zhu Z. et al.	225.8	267.62	16	138.38	105.43	111	6.7%	0.64 [0.11, 1.17]
**Subtotal (95% CI)**			**240**			**835**	**52.6%**	**0.94 [0.56, 1.32]**
Heterogeneity: $Tau^2 = 0.21$; $Chi^2 = 31.72$, df = 6 ($P < 0.0001$); $I^2 = 81\%$								
Test for overall effect: Z = 4.83 ($P < 0.00001$)								
**1.7.2 NT-proBNP Non-survivors vs. survivors**								
Chen T. et al.	1,002.43	1,072.12	113	92.33	123.43	161	9.4%	1.31 [1.04, 1.57]
Deng P. et al.	1,247.7	1,521.2	52	226.6	295.5	212	8.8%	1.41 [1.08, 1.74]
Li K. et al.	1,399	2,194	15	133.77	169.01	87	6.2%	1.50 [0.92, 2.09]
Lu Z. et al.	4,868	8,839	31	283.4	229.1	92	7.8%	1.03 [0.61, 1.46]
Nguyen A. et al.	765	1,284.11	40	266.33	469.04	247	8.7%	0.77 [0.43, 1.11]
Rath D. et al.	3,375.67	5,936.04	16	807.67	1,339.15	107	6.6%	1.05 [0.51, 1.59]
**Subtotal (95% CI)**			**267**			**906**	**47.4%**	**1.17 [0.95, 1.40]**
Heterogeneity: $Tau^2 = 0.04$; $Chi^2 = 10.18$, df = 5 ($P = 0.07$); $I^2 = 51\%$								
Test for overall effect: Z = 10.15 ($P < 0.00001$)								
**Total (95% CI)**			**507**			**1741**	**100.0%**	**1.05 [0.83, 1.28]**
Heterogeneity: $Tau^2 = 0.12$; $Chi^2 = 46.69$, df = 12 ($P < 0.00001$); $I^2 = 74\%$								
Test for overall effect: Z = 9.18 ($P < 0.00001$)								
Test for subgroup differences: $Chi^2 = 1.06$, df = 1 ($P = 0.30$), $I^2 = 5.5\%$								

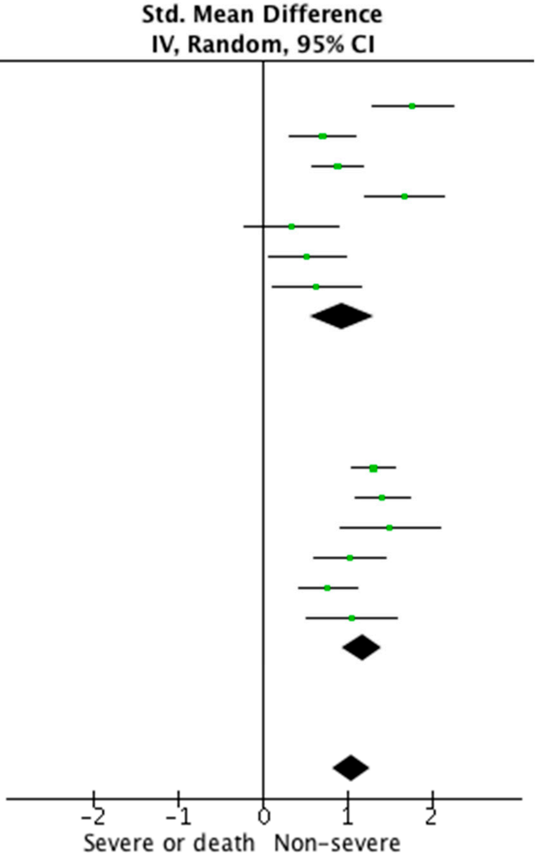

Supplementary Figure S2: Standardized mean difference and 95% confidence interval of natriuretic peptides values between patients with or without severe form of COVID-19.

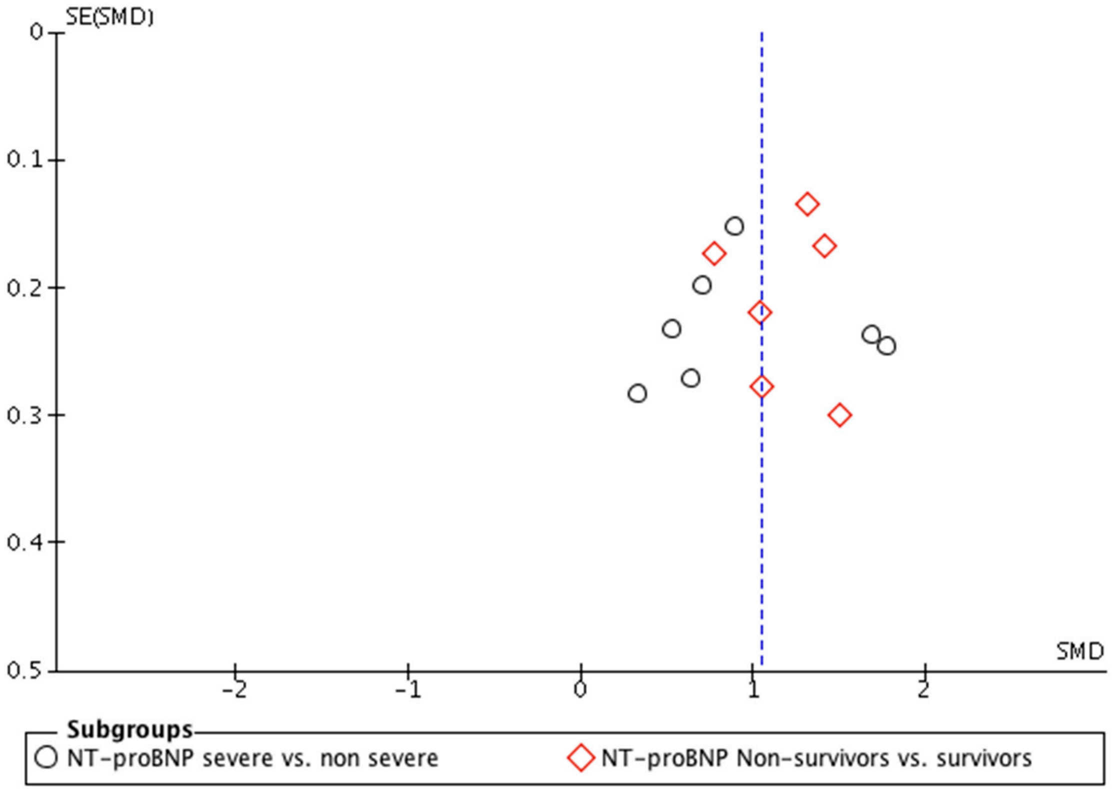

Supplementary Figure S3: Funnel plot analysis of NT-proBNP values between patients with or without severe form of coronavirus disease 2019.
